# Herbivory and Attenuated UV Radiation Affect Volatile Emissions of the Invasive Weed *Calluna vulgaris*

**DOI:** 10.3390/molecules25143200

**Published:** 2020-07-13

**Authors:** Evans Effah, D. Paul Barrett, Paul G. Peterson, Jason J. Wargent, Murray A. Potter, Jarmo K. Holopainen, Andrea Clavijo McCormick

**Affiliations:** 1 School of Agriculture and Environment, Massey University, Tennent Drive, Palmerston North 4474, New Zealand; effahevans5@gmail.com (E.E.); D.P.Barrett@massey.ac.nz (D.P.B.); J.Wargent@massey.ac.nz (J.J.W.); M.Potter@massey.ac.nz (M.A.P.); 2 Manaaki Whenua—Landcare Research, Riddet Road, Massey University, Palmerston North 4474, New Zealand; petersonp@landcareresearch.co.nz; 3 Department of Environmental and Biological Sciences, University of Eastern Finland, Yliopistonranta 1 E, FI-70210 Kuopio, Finland; jarmo.holopainen@uef.fi

**Keywords:** volatile organic compounds, plant volatiles, plant secondary metabolites, plant ecophysiology, ultraviolet radiation, biocontrol agents, heather beetle

## Abstract

*Calluna vulgaris* (heather) is an aggressive invasive weed on the Central Plateau, North Is., New Zealand (NZ), where it encounters different environmental factors compared to its native range in Europe, such as high ultraviolet radiation (UV) and a lack of specialist herbivores. The specialist herbivore *Lochmaea suturalis* (heather beetle) was introduced from the United Kingdom (UK) in 1996 as a biocontrol agent to manage this invasive weed. Like other plant invaders, a novel environment may be challenging for heather as it adjusts to its new conditions. This process of “adjustment” involves morphological and physiological changes often linked to phenotypic plasticity. The biochemical responses of exotic plants to environmental variables in their invaded range is poorly understood. The production and release of volatile organic compounds (VOCs) is essential to plant communication and highly susceptible to environmental change. This study therefore aimed to explore the VOC emissions of heather in response to different levels of UV exposure, and to feeding damage by *L. suturalis*. Using tunnel houses clad with UV-selective filters, we measured VOCs produced by heather under NZ ambient, 20% attenuated, and 95% attenuated solar UV treatments. We also compared VOC emissions in the field at adjacent sites where *L. suturalis* was present or absent. Volatiles produced by the same target heather plants were measured at four different times in the spring and summer of 2018–2019, reflecting variations in beetle’s abundance, feeding stage and plant phenology. Heather plants under 95% attenuated UV produced significantly higher amounts of (*E*)-β-farnesene, decanal, benzaldehyde, and benzeneacetaldehyde compared to 25% attenuated and ambient UV radiation. We also found significant differences in volatiles produced by heather plants in beetle-present versus beetle-absent sites on most sampling occasions. We also recorded a lower number of generalist herbivores on heather at sites where *L. suturalis* was present. Interactions between invasive plants, a novel environment, and the native communities they invade, are discussed.

## 1. Introduction

Climate change and the spread of species beyond their natural geographic boundaries are major ways in which humans have altered the environment, with consequences to species development, fitness and competitiveness [1]. Invasive plants have high phenotypic plasticity that enhances their competitiveness [2,3]. Also, new environments are unlikely to contain specialist herbivores creating enemy-poor or enemy-free spaces [4]. Hence invasive plants can allocate more resources towards competition instead of defence. Chemically, some invasive plants are known to release root exudates that are phytotoxic to natives [5,6]. However, our understanding of the chemical mechanisms behind the success of invasive plants in novel environments is still limited considering that phytochemicals mediate interactions with microbes, pollinators, herbivores and their natural enemies and plant responses to stress [7].

Plants produce volatile organic compounds (VOCs) constitutively and in response to environmental stressors [8,9,10]. VOCs mediate several interactions between organisms acting as a source of information (infochemical) to other organisms but can also act as bioactive compounds having direct impacts on surrounding species (e.g., allelopathy) [11]. The emitting plant can benefit directly from VOCs by attracting pollinators, repelling herbivores, and reducing competition by inhibiting the growth of nearby plants [11]. However, VOC emissions can also benefit the emitting plant indirectly by modifying multitrophic interactions, e.g., attracting natural enemies of their herbivores [11,12].

The blend of plant volatiles and proportions of individual compounds can be dependent on various biotic and abiotic factors. Herbivory is the most studied biotic factor in relation to plant volatiles, and it has been linked to increased emissions of individual compounds but also to unique blends that attract the natural enemies of herbivores [8,12,13,14]. For abiotic factors, the impact of temperature, soil nutrients and drought on VOCs emission has been extensively studied, while other factors such as UV radiation have received limited attention [10,15]. Nonetheless, it is known that UV radiation can influence feeding behaviour and fitness of herbivores [16,17], in addition to plant growth rates and morphology [16]. There is also evidence of UV-mediated VOC emissions, although responses may vary between volatile compounds and plants species [18,19].

The impacts of biotic and abiotic stressors on VOC emissions have been poorly studied in plant invasion scenarios and, to our knowledge, only two studies have explored the effects of environmental variables on VOC emission by invasive plants [20,21]. Under controlled conditions, elevated CO_2_ levels caused increased emissions of β-caryophyllene in the invasive weed *Mikania micrantha* [20], while poor soil fertility accounted for lower VOCs emission by the invasive *Calluna vulgaris* in the field [21]. More of these studies are needed to enhance our understanding of the mechanisms behind plant invasion, and the potential effects of an invasive plants’ volatile compounds on the native flora, fauna and microbiota.

This study aimed to explore VOC emissions of the invasive *Calluna vulgaris* (heather) in its invasive range in response to abiotic (UV radiation) and biotic (herbivory) factors. Heather is a perennial shrub in the Ericaceae family that was introduced from Europe into New Zealand’s North Is. Central Plateau by early European settlers in 1912 [22,23]. At present, it is the most widespread invasive weed in that area, outcompeting native plants and displacing their associated fauna.

In New Zealand, new heather shoots reach their final length between late November and early January, the first flowers open between mid-January and early February, and plants maintain mature photosynthetic tissue throughout the autumn and winter months (March-August) [24]. Many authors have provided detailed descriptions of the topography, soils and environmental conditions associated with heather in New Zealand [23,25,26] and a recent study documented the natural variation in heather volatile emissions on the Central Plateau [21].

Despite heather being prolific in this ecosystem, it faces two important environmental challenges. Firstly, UV radiation levels in New Zealand are considerably higher when compared to heather’s native range in Europe [27], which may induce changes to the plant’s biochemistry [28]. Secondly, heather beetle (*Lochmaea suturalis*) was introduced as a biocontrol agent into Tongariro National Park in 1996 and it is starting to have a significant impact on heather density [29,30].

We investigated the volatile production of heather in response to these abiotic and biotic factors; i.e., exposure to UV radiation in tunnel house conditions and feeding damage by *L. suturalis* under natural field conditions. To investigate the impact of UV on VOC emissions, heather plants of similar size and phenology were collected from the North Is. Central Plateau, maintained under the same outdoor conditions for six weeks and then randomly allocated to New Zealand (NZ) ambient, 20% attenuated or 95% attenuated UV screened tunnel house conditions. VOCs were measured from treatment plants after 75 days of UV exposure.

In the field study, VOC measurements were made at four different sampling times in two adjacent sites where the heather beetle was present or absent. VOCs were collected when (1) adult *L. suturalis*, (2) first instar *L. suturalis*, (3) third instar and adult *L. suturalis* and (4) newly emerged adult *L. suturalis* were dominant at the beetle-present site. These sampling times reflected the plant’s responses during the different phenological states of heather and abundance of the herbivore. We also collected all arthropods from the heather plants, to estimate herbivore load and how the distribution of other species might be affected by the presence of *L. suturalis.* Based on existing literature, we hypothesised that volatile emissions will be higher for plants exposed to high levels of UV radiation and beetle damage. We also expected to observe variation in the arthropod community composition between sites where *L. suturalis* was present or absent, likely due to changes in plant food quality, increased competition or induced plant defences.

## 2. Results

### 2.1. Volatile Organic Compounds Emissions under UV Treatments

**Figure 1:**
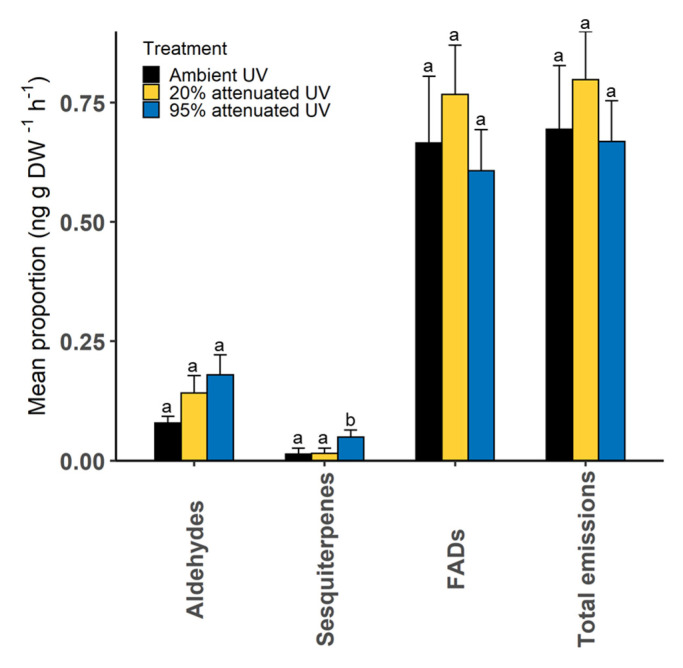
Proportions of major chemical classes in heather plants exposed to ambient and attenuated UV (n = 10 for each treatment). Y-axis shows log10x + 1 transformed mean ± SE proportion of compound classes. Different letters indicate significant differences.

After 75 days of UV exposure, foliar volatile compounds produced by heather plants exposed to ambient, 20% attenuated and 95% attenuated UV were collected using the “push-pull” headspace sampling technique. Compounds were grouped into major chemical classes (Table S3) and compared between the treatments. Except for sesquiterpenes (Kruskal–Wallis; *X*^2^ = 7.74, df = 2, *p* = 0.021), the proportions of major VOC classes did not differ between the treatments, although fatty acid derivatives (FADs) and total volatile emissions were marginally higher for plants exposed to 20% attenuated UV (Figure 1).

**Figure 2:**
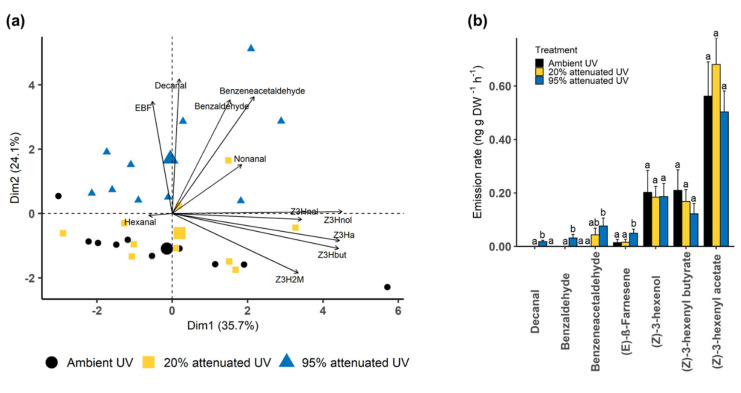
(**a**) Principal component analysis (PCA) biplot based on the volatile compounds produced by heather under ambient, 20% attenuated and 95% attenuated UV. (**b**) The relative proportion of compounds with higher contributions to PC1 and PC2 between the three UV treatments (n = 10). *Y*-axis of barplot (**b**) shows log10x + 1 transformed mean ± SE emission rate compounds and different letters indicate significant differences. Abbreviations in PCA: (*E*)-β-farnesene (EBF), (*Z*)-3-hexenal (Z3Hnal), (*Z*)-3-hexenol (Z3Hnol), (*Z*)-3-hexenyl acetate (Z3Ha), (*Z*)-3-Hexenyl butyrate (Z3Hbut), (*Z*)-3-hexenyl 2-methylbutyrate (Z3H2M).

Principal component analysis (PCA) was performed based on all the volatile compounds identified from heather plants under each UV treatment. Results of the PCA show separation between the VOCs produced by heather exposed to 95% attenuated and ambient UV. The first and second principal components (PC1 and PC2) explained 59.8% of the variation in emissions between the three treatments. PC1 was characterised by the fatty acid derivatives (*Z*)-3-hexenol, (*Z*)-3-hexenyl acetate and (*Z*)-3-hexenyl butyrate. PC2 was characterised by aldehydes benzeneacetaldehyde, benzaldehyde, decanal and a sesquiterpene (*E*)-β-farnesene (Figure 2a). 

The relative proportions of compounds with higher contributions to PC1 and PC2 (Figure S1) were compared between the three UV treatments (Figure 2b). The results show significant differences in the proportion of (*E*)-β-farnesene (Kruskal–Wallis; *X*^2^ = 7.74, df = 2, *p* = 0.021), benzaldehyde (Kruskal–Wallis; *X*^2^ = 17.25, df = 2, *p* < 0.001), benzeneacetaldehyde (Kruskal–Wallis; *X*^2^ = 11.96, df = 2, *p* = 0.003) and decanal (Kruskal–Wallis; *X*^2^ = 23.84, df = 2, *p* < 0.001) with higher emissions from heather under 95% attenuated UV (Figure 2b).

### 2.2. Herbivory Experiment

#### 2.2.1. Plant and *L. suturalis* Phenology during the Experiment

The phenology of *L. suturalis* and heather plants were visually inspected during each sampling period. On 14 November 2018, the plants at the site where *L. suturalis* was present were reasonably healthy but showing sign of previous damage and not as lush and green as the control site. During the second sampling on 11 December 2018, any new season’s growth on plants at this site had been chewed by the beetle. They were showing signs of desiccation while control plants remained undamaged with much newer season’s growth evident. On 31 January 2019, plants at the beetle-present site remained desiccated, showed increased browning and little sign of flowering while control site plants displayed a profusion of flowers. During the fourth sampling on 25 March 2019, these plants were very brown and desiccated while control plants were green with matured current season growth.

**Figure 3:**
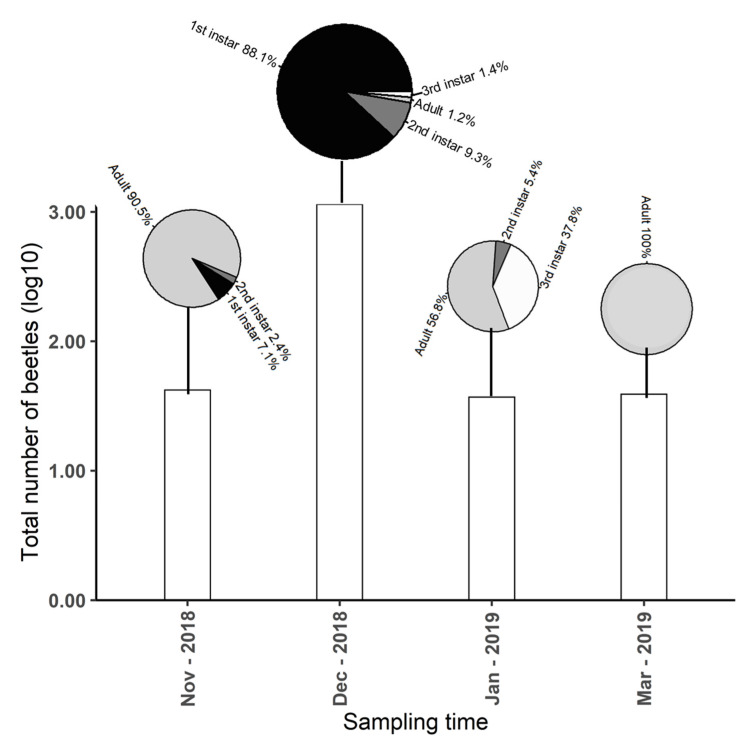
Developmental stage and abundance of *Lochmaea* *suturalis* during the four volatile organic compound (VOC) measurements periods. Bars indicate the total number of *L. suturalis* present and pie charts show the proportion of each developmental stage at a given sampling time.

*L. suturalis* also progressed through different developmental stages at the beetle-present site during the sampling period. During the first collection on 14 November 2018, adult *L. suturalis* were most abundant. On 11 December 2018, during the second sampling, collections were dominated by first instar *L. suturalis*. During the third sampling on 31 January 2019 third instar larvae and adult *L. suturalis* were more abundant. During the fourth and final sampling on 25 March 2019 the beetle-present site was again dominated by newly emerged adult *L. suturalis* (Figure 3). The control plants (beetle-absent) remained beetle free for the entire duration of the experiment.

#### 2.2.2. Volatile Profiles of Heather in Beetle-present and Beetle-Absent Sites

**Figure 4:**
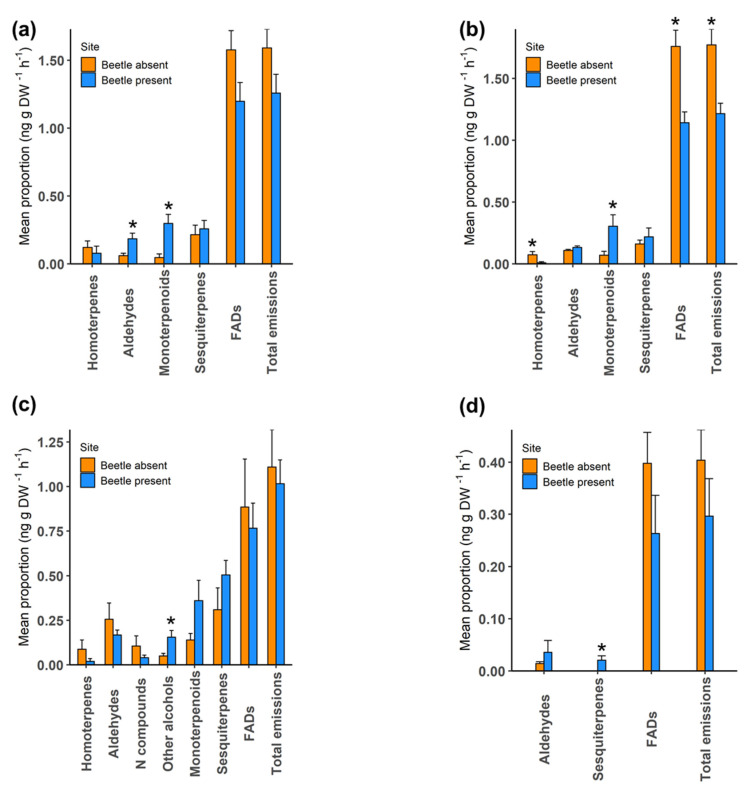
Comparison of major chemical classes between sites at (**a**) 14 November 2018, (**b**) 11 December 2018, (**c**) 31 January 2019 and (**d**) 25 March 2019. Y-axis shows log10x + 1 transformed mean ± SE proportion of chemical classes and asterisk indicate a significant difference. n = 7 for the beetle-present site in (**c**), otherwise 8 replicates per treatment at each sampling time. Asterisk indicates significant differences (* *p* < 0.05).

During the four sampling times, volatile organic compounds in the headspace of enclosed heather foliage were measured and compared between the two study sites. First, we compared the proportion of major chemical classes (Table S3) produced by heather at areas where the heather beetle was present and absent. Overall, the results reveal variations in some chemical classes between sites at each sampling time (Figure 4).

In November 2018, aldehydes (Wilcoxon sum rank test; *p* = 0.024) and monoterpenoids (Wilcoxon sum rank test; *p* = 0.005) were produced in significantly higher amounts at the beetle-present site when the site was dominated by adult *L. suturalis* and plant foliage was budding despite signs of damage (Figure 4a).

In December 2018, when foliage at the beetle-present site was showing signs of beetle grazing and desiccation, with first and second instar *L. suturalis* being dominant, the proportion of fatty acid derivatives (Wilcoxon sum rank test; *p* = 0.005), monoterpenoids (Wilcoxon sum rank test; *p* = 0.029), homoterpenes (Wilcoxon sum rank test; *p* = 0.040) and total VOC emissions (Wilcoxon sum rank test; *p* = 0.005) varied significantly between the sites (Figure 4b). 

In January 2019, only the proportion of alcohols differed significantly between the sites (Wilcoxon sum rank test; *p* = 0.043) when plants at the beetle-absent site were heavily flowering while third instar and adult *L. suturalis* were dominant on the desiccated poorly flowering heather at the site where the beetle was present. The proportion of monoterpenoids was higher at the beetle-present site, but this was not significant (Figure 4c).

In March 2019, except for sesquiterpenes (Wilcoxon sum rank test; *p* = 0.032), there were no significant differences in the major chemical classes between sites in VOCs measured, despite foliage at the beetle-absent site being green and mature as compared to the brown desiccated plants at the site where newly emerged adult *L. suturalis* were present (Figure 4d).

**Figure 5:**
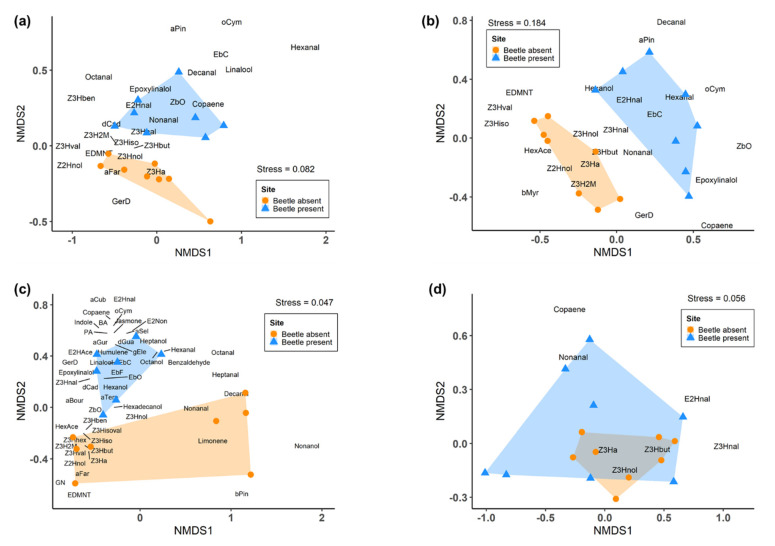
Non-metric multidimensional scaling (NMDS) plots showing the first (NMDS1) and second (NMDS2) axes of VOCs identified from heather at beetle-present and beetle-absent sites in (**a**) November 2018, (**b**) December 2018, (**c**) January 2019 and (**d**) March 2019. n = 7 for the beetle-present site in (c), otherwise 8 replicates per treatment were done in all sampling times. **Abbreviations:** (*Z*)-3-hexenyl benzoate (Z3Hben), δ-cadinene (dCad), (*E*)-2-hexanal (E2Hnal), (*Z*)-3-hexenyl 2-methylbutyrate (Z3H2M), (*Z*)-3-hexenyl valerate (Z3Hval), (*E*)-4,8-dimethyl-1,3,7-nonatriene (E-DMNT), (*Z*)-2-hexenol (Z2Hnol), germacrene D (GerD), (*E*,*E*)-α-farnesene (aFar), (*Z*)-3-hexenyl acetate (Z3Ha), (*Z*)-3-hexenol (Z3Hnol), (*Z*)-3-hexenal (Z3Hnal), (*Z*)-3-hexenyl butyrate (Z3Hbut), (*Z*)-3-hexenyl isobutyrate (Z3Hiso), (*Z*)-β-ocimene (Zbo), α-pinene (aPin), (*E*)-β-caryophyllene (EbC), o-cymene (oCym), hexyl acetate (HexAce), β-myrcene (bMyr), (*E*)-β-farnesene (EBF), (*Z*)-3-hexenyl hexanoate (Z3Hhex), (*Z*)-3-hexenyl isovalerate (Z3Hisoval), geranyl nitrile (GN), phenylethyl alcohol (PA), α-bourbonene (aBour), α-cubebene (aCub), benzyl alcohol (BA), α-gurjunene (aGur), α-selinene (aSel), α-terpineol (aTerp), β-pinene (bPin), γ-elemene (gEle), δ-guaiene (dGua), (*E*)-β-ocimene (EbO), (*E*)-2-hexenyl acetate (E2HAce), (*E*)-2-nonenal (E2Non).

Permutational multivariate analysis of variance (PERMANOVA) was used to compare the overall volatile profile of heather plants at the beetle-present and beetle-absent sites, and the patterns in emission visualised using non-metric multidimensional scaling (NMDS). We found variation in volatile profiles between heather from beetle-present and beetle-absent sites in November 2018 (PERMANOVA; Pseudo-*F*_1,14_ = 3.21, *p* = 0.028). The similarity percentage analysis revealed that the homoterpene (*E*)-DMNT, six fatty acid derivatives, three sesquiterpenes and monoterpene (*Z*)-β-ocimene were the main compounds contributing to the observed variation (Figure 5, Figure S2).

Heather’s volatile profiles also varied between sites in December 2018 (PERMANOVA; Pseudo-*F*_1,14_ = 6.66, *p* = 0.006). Fatty acid derivatives (*Z*)-3-hexenyl acetate, (*Z*)-3-hexenol, (*Z*)-3-hexenal, (*Z*)-3-hexenyl butyrate, (*Z*)-3-hexenyl 2-methylbutyrate and terpenes (*Z*)-β-ocimene, germacrene D, (*E*)-β-caryophyllene were the main compounds accounting for the observed differences (Figure 5, Figure S2).

Similar segregation was observed in the composition of heather’s volatile profile between sites in January 2019 (PERMANOVA; Pseudo-*F*_1,13_ = 3.55, *p* = 0.022). A total of 54 volatile compounds were identified from plants at beetle-present and beetle-absent sites. The similarity percentage analysis revealed that 24 compounds were the main drivers of the observed pattern in VOC emissions between sites (Figure 5, Table S2).

Unlike the other sampling times, volatile profiles did not differ between heather at beetle-present and beetle-absent sites in March 2019 (PERMANOVA; Pseudo-*F*_1,14_ = 2.14, *p* = 0.112, Figure 5).

### 2.3. Arthropod Community Composition between Sites

**Figure 6:**
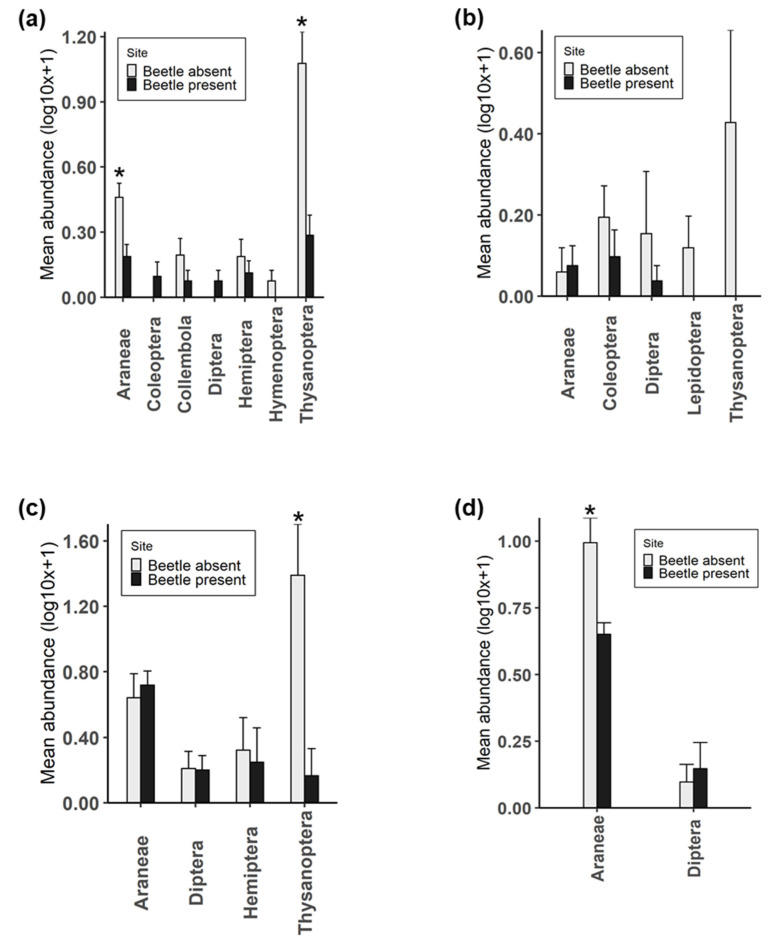
Comparing the abundance of arthropods other than *L. suturalis* on heather between sites. Samples collected on (**a**) November 2018, (**b**) December 2018, (**c**) January 2019 and (**d**) March 2019 using the beating tray technique (n = 3). Bars show mean ± SE and asterisk indicates a significant difference between sites. Asterisk indicates significant differences (* *p* < 0.05).

During each sampling period, arthropods present on heather plants were collected using the beating tray technique. Collected specimens were identified to order and compared between beetle-present and beetle-absent sites. Overall, there were differences in the arthropod composition (excluding *L. suturalis*) on heather plants between beetle-present and beetle-absent sites (Figure 6). The results show significant differences between sites for the number of Araneae collected in November 2018 (Wilcoxon sum rank test; *p* = 0.012) and March 2019 (Wilcoxon sum rank test; *p* = 0.008) with higher numbers at the site where *L. suturalis* was absent (Figure 6a,d). Similarly, higher numbers of Thysanoptera were recorded at the site where *L. suturalis* was absent in November 2018 (Wilcoxon sum rank test; *p* = 0.003) and January 2019 (Wilcoxon sum rank test; *p* = 0.009) (Figure 6a,c). The number of Thysanoptera was only marginally (Wilcoxon sum rank test; *p* = 0.076, Figure 6b) higher at the site where *L. suturalis* was absent in December 2018.

## 3. Discussion

### 3.1. UV Mediated Volatile Emissions 

Plant responses to UV radiation differ between species, genotypes and even sex [31,32,33], but generally, exposure to elevated UV elicits several adaptive mechanisms in plants, particularly the production of secondary metabolites, including UV absorbing compounds and antioxidants [34,35,36]. Some plants may increase growth and productivity in response to higher UV exposure [37,38], although adverse outcomes have also been documented [39,40]. UV radiation also induces the production of other chemicals in plants, including VOCs [32,41]. The effect of UV exposure on heather’s volatile emissions has not been previously documented, but there is evidence of a reduction in the concentration of the amino acid isoleucine, which is a precursor for many VOCs including aldehydes and esters [9], in response to enhanced UV-B [42].

Results from the present study demonstrate that long term exposure to NZ ambient UV affects the level of VOC’s emitted by heather. Compared to NZ ambient UV (highest UV level), exposure to 95% attenuated UV was associated with higher emissions of sesquiterpenes such as (*E*)-β-farnesene and some aldehydes like benzaldehyde and benzeneacetaldehyde. In agreement with our results, Machado and coworkers [43] found that volatile emissions of juvenile *Eucalyptus globulus* also decreased after two days of exposure to elevated UV-B, with reductions in terpenes and aldehydes. The amount of monoterpenes including α-phellandrene, α-thujene and o-cymene decreased significantly in UV-B exposed plants, while aldehydes (particularly benzaldehyde) showed an initial increase after stress removal but decreased after that [43]. Similar studies report a reduction in total terpenes by *Pistacia lentiscus* in response to elevated UV addition [44] and the enhanced production of amino acid-derived volatiles by attenuated UV radiation in *Vitis vinifera* [45].

Contrary to the above, a number of studies have reported increased VOC emissions under elevated UV radiation [32,46,47,48]. An increase in VOC emissions under elevated UV radiation, particularly UV-B, has been suggested as a plant defensive mechanism to protect tissues from adverse effects [8,49]. The discrepancies between studies may be a result of species-specific responses to UV radiation, but the effect likely varies depending on the duration and intensity of UV exposure. For instance, emission of terpenoids by indoor-grown Norway spruce (*Picea abies*) after 4 h of UV-B exposure showed an increased amount of bornyl acetate, borneol, myrcene, and limonene within the first three days but emissions returned to normal after three weeks of treatment [50]. In contrast, needle monoterpene and sesquiterpene emission of field-grown Norway spruce seedlings did not respond to continuously enhanced UV-B radiation up to 30% above the ambient level [51].

Another factor to consider may be the initial state of the plant. In our study, plants were collected from the field, where they had been exposed (and possibly adapted) to high UV conditions prior to the experiment. Hence, it is feasible that they would react differently to plants grown in a greenhouse or under low UV conditions. We, therefore, encourage future studies to investigate whether a reduction in metabolites is typical behaviour for this species under elevated UV radiation, or if changes in VOC emissions are context-dependent.

### 3.2. Damage by Specialist Herbivore and VOC Emissions

Herbivore damaged plants produce volatile blends that differ qualitatively or quantitatively from those of undamaged plants. Herbivore-induced plant volatiles can be released locally from the damaged site or systemically, from undamaged parts of attacked plants; as well as by nearby primed plants [13,52,53]. These herbivore-induced VOCs can play a critical role in plant defence against enemies [8,13]. Our results suggest that heather plants change their volatile blends in response to attack by the specialist herbivore, *L. suturalis*. The results also indicate that heather’s response to feeding damage by *L. suturalis* varies with the abundance of the beetle, its feeding stage and the plant’s phenology. To our knowledge, this is the first report of a plant’s VOC response to feeding by its specialist herbivore across different plant phenological stages under natural conditions.

Several studies indicate that lipoxygenase products, including the C_6_ green leaf volatiles, are readily released from broken tissues and constitute a large percentage of the VOCs emitted by plants attacked by chewing herbivores [8,54]. However, feeding damage by *L. suturalis* did not cause higher emission of this group of compounds in our study. Perhaps this is because plants in the field are subject to previous and continuous damage, unlike plants in controlled laboratory studies that are naïve to herbivore damage [55]. In fact, higher emissions of the green leaf volatiles (*Z*)-3-hexenyl acetate, (*Z*)-3-hexenyl 2-methylbutyrate, (*Z*)-3-hexenol and (*Z*)-3-hexenyl butyrate were instead found at the site where *L. suturalis* was not present.

On the other hand, terpenoids, whose production incurs high raw material and enzymatic costs compared to other compounds [56], were emitted in higher amounts at the beetle-present site. Emissions of (*Z*)-β-ocimene, (*E*)-β-caryophyllene, (*E*)-β-farnesene, δ-cadinene and δ-guaiene were significantly higher at the site where *L. suturalis* was present (Table S2 and Figure S2), suggesting a possible selective defence by heather against generalist and specialist herbivores or production of these compounds being dependant on the level of herbivory. The beetle-absent site was not herbivore-free as evidenced by the number of thrips and lepidopteran larvae caught at this site. Besides, the higher emissions of homoterpenes and fatty acid derivatives from heather at the beetle free site may indicate damage by thrips. Also, VOC profiles changed over time in relation to abundance and growth stages of *L. suturalis*, suggesting that plant responses can be quite specific to the identity and developmental stage of the attacker. This supports earlier reports indicating that the composition of volatiles emitted by attacked plants can depend on the stage, abundance and feeding pattern of herbivores [57,58,59,60].

We also acknowledge that the phenology and physiology of target plants at sampling times had a significant impact on the composition of measured plant volatiles. Our results show that the differences between VOC profiles of plants at beetle-present and beetle-absent sites were more marked at the leaf budding and flowering stage and negligible past the flowering stage. This indicates that young and reproductive tissue are better defended, as evidenced by their higher VOCs emissions than mature non-reproductive organs [61,62].

### 3.3. Potential Ecological Impact of Variable Emissions

Changes in VOC emissions by heather in response to abiotic or biotic factors could have different effects on other community members, and may also directly or indirectly affect the emitting plant. Most attacked plants can reduce herbivore loads by producing volatile compounds that can directly repel herbivores or modify the interaction between herbivores and their natural enemies [8,12,63]. We did not see any evidence of increased predation at the sites where *L. suturalis* was present but rather a displacement of other herbivores. Specialist herbivores like *L. suturalis* may be less negatively impacted by the defences built up by their host plant compared to generalist [64], which may explain the lower abundance of other arthropods especially thrips and lepidopteran larvae at site where *L. suturalis* was present. However, the variation in arthropod communities between the beetle absent or present sites could also be attributed to changes in the quality and quantity of the plant food source. Generalist herbivores may avoid heather plants attacked by *L. suturalis* because food is less available or assimilable due to the induction of chemical defences.

A review exploring the effects of changing environmental factors on tritrophic interactions found that VOC emissions induced by some abiotic factors can influence the communication between plants, herbivores and the natural enemies of herbivores, but this may vary between stressors and organisms [10]. In plant invasion scenarios, changes in the amount and composition of plant VOCs in response to stresses could impact plant competition between native and invasive plants by interfering with or interrupting signals between neighbouring plants and other organisms or by disrupting communication altogether. This phenomenon has not yet been studied, but such information is vital to broaden our understanding of plant invasions, particularly in the context of climate change, since abiotic factors such as temperature, drought and CO_2_ are expected to rise in the coming years [65].

In addition to the potential disruption of multitrophic interactions, compounds emitted under stressful conditions may directly affect nearby native plants, but to our knowledge, there is only one study exploring such effects within a plant invasion context [20]. The authors showed that elevated CO_2_ levels cause increased emissions of the volatile compound β-caryophyllene in one of the worst invasive weeds in the world (*Mikania micrantha*), which was linked to enhanced phytotoxicity and allelochemical properties against various potential competitors. This is also a research area that requires further attention.

Finally, the production of plant volatiles can have varying costs on raw materials and biosynthetic enzymes, and plants may reduce such costs by utilising individual compounds in multiple roles or catabolising compounds that are not needed [56]. There may also be a trade-off between the emission of plant volatiles and the use of those resources for growth and reproduction [66,67], which could be important for invasive species like heather that outcompete native plants through rapid growth and high reproduction rates. Therefore, it is not surprising that some stressors may have adverse effects on VOC production and emission by invasive plants.

## 4. Materials and Methods

### 4.1. Experimental Design

#### 4.1.1. UV Experiment

Heather plants of similar size were collected from a natural population at Taurewa (Long. 175.556591–Lat. −39.081535) on the Central Plateau, North Island, New Zealand, during September 2018 and transplanted into plastic pots (23 cm × 26 cm) maintaining the root-bound soil intact. 

The plants were maintained outdoors at Massey University Plant Growth Unit, Palmerston North, New Zealand (Long. 175. 614,600–Lat. −40.377474) for six weeks, then on 1 November transferred to a tunnel house facility and maintained under one of the three randomly allocated treatments. Treatment one (control) was unscreened ambient light. Treatment two used a polyethylene UV-modifying filter which achieved > 95% attenuation of ambient UV (“UV-opaque”) (Lumisol “019”, BPI Visqueen, UK). Treatment three achieved 20% attenuation of ambient UV (“UV-transparent”) (Lumisol “018”, BPI Visqueen, UK). The UV-modifying filters are described in more detail in [68]. Although some variation in Photosynthetically Active Radiation (PAR: 400–700 nm) was recorded under the films at the date of sampling (Table S1), these differences were not significant and were likely due to variable cloud cover at the time of measurement, only UV-A and UV-B measurements varied significantly between the films (Table S1).

A dividing curtain of Lumisol “019” was installed at the junction of the two screen types to prevent UV leakage between treatments. Plants were watered using individual drip irrigation for three minutes, four times daily, with no additional nutrients. To maintain more equitable tunnel house temperatures, fans were installed and programmed to run at a setpoint of 20 °C. Temperature and humidity were recorded continuously using HOBO data loggers (HOBO Pro v2, Onset, Pocasset, MA, USA) (Table 1), and the plants were exposed to these treatments for 75 days. For VOC collection plants were transferred directly from the tunnel house into a temperature-controlled room (25 °C) under continuous lighting, and volatiles collected on the same day. 

#### 4.1.2. Herbivory Experiment

The field-based herbivory experiment was conducted during summer (November 2018 to March 2019). Two adjacent sites, 880 m a.s.l., and of the same volcanic soil type were identified within the Waiouru Military Training Area on the Central Plateau, North Island, New Zealand (Treatment site Long. 175.687533–Lat. −39.406130, Control site Long. 175.685303–Lat. −39.431929). At the time of the experiment, the biocontrol agent, *L. suturalis* was present at one of the sites but absent from the other. Volatiles from eight target plants were measured in each treatment. 

At the initial setup, and during the sampling period, the number and stage of beetles at each of the sites was determined by sampling with the beating tray technique, as described by [21] on three plants adjacent to the targeted VOC sampling plant. Collected heather beetles and other invertebrate specimens were preserved in 70% ethanol and later classified to order.

To elucidate heather’s response to feeding damage caused by *L. suturalis* in combination with different plant phenological stages, volatiles and invertebrates were collected on four different occasions on 14 November 2018, 11 December 2018, 31 January 2019 and 25 March 2019 during sunny and dry weather conditions.

### 4.2. VOCs Sampling

For both UV-B and herbivory experiments, foliar volatiles from heather plants were collected using the “push-pull” headspace sampling technique [69] and analysed following the same protocol as in [21]. Heather foliage was enclosed in a new oven bag, and carbon filtered air was pushed into the bag (1.70 L/min) and simultaneously pulled out (1.20 L/min) through PTFE tubes using a PVASS22 pump. VOCs in the air surrounding the enclosed foliage were collected using a volatile collection trap with 30 mg HayeSep Q adsorbent medium (Volatile Assay Systems Rensselaer, NY, USA). The collection traps were eluted using 200 μL of 99% hexane (Sigma Aldrich, St Louis, MO, USA) with 10 ng/mL nonyl acetate (C_11_H_22_O_2_) (Sigma Aldrich, Buchs, Switzerland) and analysed using gas chromatography coupled to mass spectrometry (GC-MS) (QP2010; GCMS Solution version 2.70, Shimadzu Corporations, Kyoto, Japan), with a 30 m × 250 μm × 0.25 μm TG-5MS capillary column (Thermo Fisher Scientific, Waltham, MA, USA). 

Operating conditions of the GC-MS and identification of compounds followed the protocol described in [21]. The carrier gas was helium and was supplied at pressure 53.5 kPa, total flow 14.0 mL/min, linear velocity 36.3 cm/s and purge flow 3.0 mL/min. The temperature programme was: initial oven temperature 50 °C, held for 3 min, rising at 5 °C/min to 95 °C and increased to 145 °C at a rate of 15 °C/min; injection temperature 230 °C; injection volume 1 μL, split mode; split ratio 10:1. Chromatographic analyses were performed in full-scan mode and area of targeted peaks quantified relative to that of the internal standard.

### 4.3. Data Analysis

UV response data were square root transformed and analysed using Principal component analysis (PCA). PCA was performed using the “FactoMiner” package [70]. Individual compounds with higher contributions to the first and second principal components were selected for subsequent analyses.

The volatile profiles of heather plants from sites where *L. suturalis* was present or absent were compared using permutational multivariate analysis of variance (PERMANOVA) [71,72]. PERMANOVA was run using the “adonis” function in the “vegan” package R [73]. When groups were significantly different, the similarity percentage (SIMPER) was used to identify compounds contributing to the differences [72]. The patterns in VOC emissions between groups were visualised using non-metric multidimensional scaling (NMDS), also with the “vegan” package. Both PERMANOVA and NMDS were based on Bray-Curtis dissimilarities using square root transformed VOCs data. All statistical analyses were performed using R.

Major volatile classes and the relative proportion of individual compounds selected through PCA were compared between the groups using the Kruskal–Wallis or Wilcoxon rank-sum test. Arthropod abundance between sites was also compared using the Wilcoxon rank-sum test. For this comparison, *L. suturalis* was excluded.

## 5. Conclusions

This study shows that both abiotic (UV radiation) and biotic (herbivory) factors can influence volatile emissions of an invasive species. Elevated UV-radiation caused a significant reduction in sesquiterpene emissions when compared to the 95% attenuation treatment. The presence of the specialist herbivore (*L. suturalis*) was associated with increased emissions of mono and sesquiterpenoids, aldehydes and some alcohols, and reduced emissions of fatty acid derivatives and homoterpenes. However, the effects varied across the sampling season reflecting changes on the developmental stage of the herbivore, plant phenology and herbivore abundance.

Contrary to our initial prediction, elevated UV radiation and herbivory did not always cause an increase in emission of the major VOC groups. We observed, for instance, a significant decrease in terpenoid emission in relation to elevated UV radiation and fatty acid derivatives in response to herbivore damage. These results differ from those of most laboratory-based studies. A plausible explanation is that previous exposure to environmental factors (such as UV in tunnel house studies) and the presence of previous damage or multiple stressors under field conditions (in herbivory trials) may result in different outcomes from studies under controlled settings using naïve plants and a single stress factor. This highlights the need for more studies under field scenarios or using field-collected plants.

This paper also discusses how VOCs could influence the competitive outcomes between native and invasive species, either directly by acting as allelochemicals or indirectly by affecting the plant’s interactions with microbes, herbivores and higher trophic levels. Therefore, investigating their emission and the environmental factors influencing it can be useful to support biocontrol and management strategies.

## Figures and Tables

**Table 1:** Microclimatic measurements for tunnel house and ambient treatments.

	Treatment (Mean ± SD)		
**Variable**	**Ambient**	**20% Attenuation**	**95% Attenuation**
**Temperature (°C)**	18.56 ± 3.96	21.98 ± 5.76	22.17 ± 5.59
**Humidity (%)**	74.76 ± 13.15	64.19 ± 15.49	64.73 ± 16.09

## Supplementary Materials

The following are available online at https://www.mdpi.com/1420-3049/25/14/3200/s1, Figure S1: Contributions of variables in PCA based on VOCs identified from heather under different UV-B levels, Figure S2. Comparison of main compounds contributing to the observed differences in emissions between sites selected through SIMPER for samples collected on (a) November 2018 and (b) December 2018. Table S1. Photosynthetically active radiation (PAR), UV-A and UV-B measured under the films. Table S2. VOCs selected through SIMPER as the main compounds contributing to the variations in volatile profiles of heather at second and third instar *L. suturalis* for beetle-present and beetle-absent sites in January 2019. Table S3. List of compounds identified from the headspace of heather during the herbivory and UV experiments.
