# Supplementary material for: Herbivory and Attenuated UV Radiation Affect Volatile Emissions of the Invasive Weed Calluna vulgaris
Source: Molecules. 2020 Jul 13;25(14):3200. doi: 10.3390/molecules25143200 (PMC7397131; doi:10.3390/molecules25143200)
Supplement: Supplementary file 1 [file molecules-25-03200-s001.pdf]

Supplementary material

Table S1. Photosynthetically active radiation (PAR), UV-A and UV-B measured under the films

Variable	20% attenuation	95% attenuation	T-test
**PAR (µmol m^{-2} s^{-1})**	629 ± 295	933 ± 437	*P* = 0.233
**UV-A (uW cm^{-2})**	1513 ± 617	63 ± 17	*P* < 0.001
**UV-B (uW cm^{-2})**	117 ± 42	41 ± 25	*P* = 0.008

Table S2. VOCs selected through SIMPER as the main compounds contributing to the variations in volatile profiles of heather at second and third instar *L. suturalis* infested and non-infested sites in January 2019. Table shows mean ± SE emission rate (ng gDW^{-1} h^{-1}) of compounds. *P*-values calculated using the Wilcoxon sum rank test and bold fonts indicate compounds that were significantly different between sites (n = 7 for beetle present and 8 for beetle absent)

	**Emission rate (mean ± SE)**		
**Compound**	**Beetle absent**	**Beetle present**	***P*-value**
(*E*)-DMNT	0.300 ± 0.210	0.049 ± 0.043	0.396
(*Z*)-2-hexenol	0.497 ± 0.309	0.118 ± 0.065	0.583
(*Z*)-3-hexenol	0.613 ± 0.218	0.544 ± 0.310	0.779
(*Z*)-3-hexenyl 2-methylbutyrate	0.857 ± 0.554	0.298 ± 0.097	0.770
(*Z*)-3-hexenyl acetate	13.280 ± 5.884	2.842 ± 1.155	0.779
(Z)-3-hexenyl benzoate	0.265 ± 0.182	0.173 ± 0.055	0.381
(*Z*)-3-hexenyl butyrate	5.723 ± 4.014	2.093 ± 0.748	0.601
(*Z*)-3-hexenyl valerate	0.527 ± 0.346	0.203 ± 0.072	0.768
(*Z*)-β-ocimene	0.179 ± 0.082	0.977 ± 0.619	0.115
**(*E*)-β-caryophyllene**	**0.081 ± 0.059**	**0.520 ± 0.226**	**0.008**
**Copaene**	**0.000 ± 0.000**	**0.390 ± 0.134**	**0.007**
Decanal	0.399 ± 0.208	0.087 ± 0.020	0.779
Epoxylinalol	0.043 ± 0.043	0.167 ± 0.057	**0.058**
Geranyl nitrile	0.362 ± 0.206	0.035 ± 0.030	0.415
Germacrene D	0.113 ± 0.072	0.573 ± 0.377	0.380
Linalool	0.000 ± 0.000	0.587 ± 0.369	**0.057**
Nonanal	0.592 ± 0.233	0.264 ± 0.061	0.536
Octanal	0.082 ± 0.059	0.053 ± 0.010	0.107
**Phenylethyl alcohol**	**0.000 ± 0.000**	**0.171 ± 0.067**	**0.002**
α-bourbonene	0.118 ± 0.059	0.178 ± 0.112	0.950
(*E*,*E*)-α-farnesene	1.280 ± 0.602	0.109 ± 0.052	0.502
**(*E*)-β-farnesene**	**0.025 ± 0.024**	**0.072 ± 0.009**	**0.014**
**δ-cadinene**	**0.042 ± 0.026**	**0.249 ± 0.065**	**0.022**
**δ-guaiene**	**0.000 ± 0.000**	**0.249 ± 0.063**	**0.001**

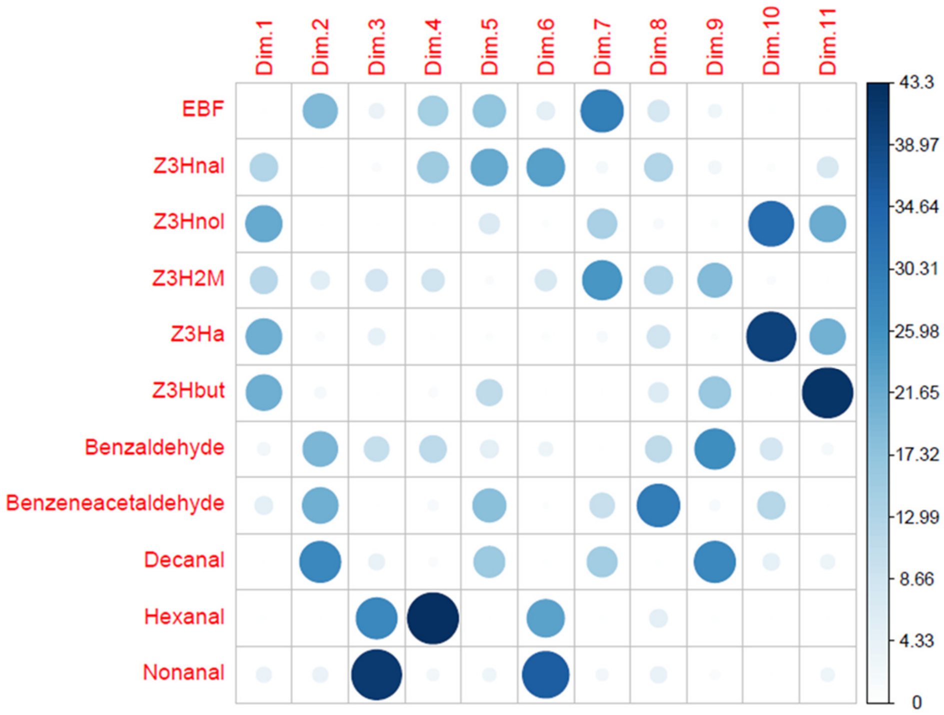

Figure S1. Contributions of variables in PCA based on VOCs identified from heather under different UV-B levels (n = 10 for each treatment). **Abbreviations**: (*E*)-β-farnesene (EBF), (*Z*)-3-hexenal (Z3Hnal), (*Z*)-3-hexenol (Z3Hnol), (*Z*)-3-hexenyl acetate (Z3Ha), (*Z*)-3-Hexenyl butyrate (Z3Hbut), (*Z*)-3-hexenyl 2-methylbutyrate (Z3H2M)

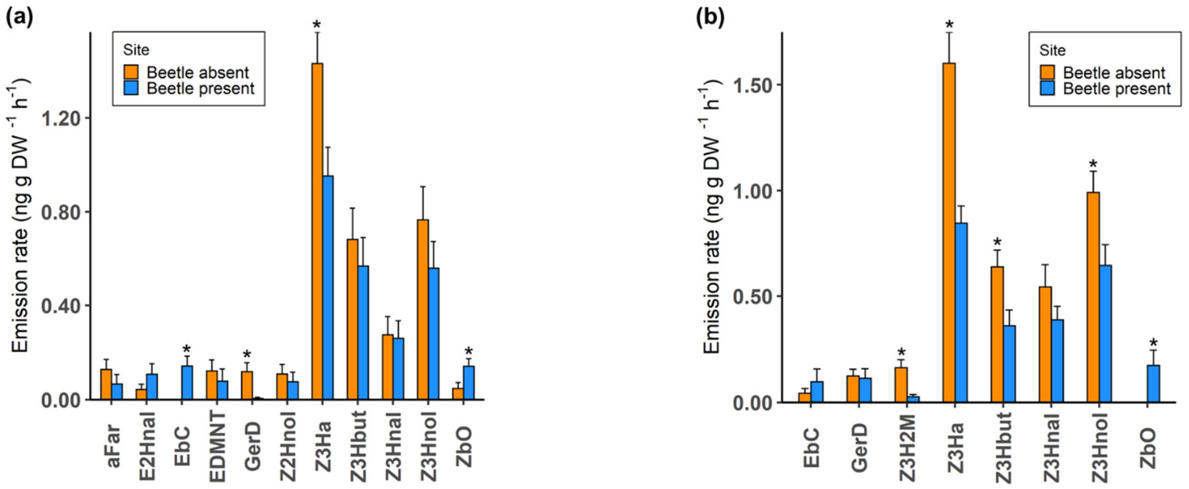

Figure S2. (a) NMDS plot for VOCs identified from heather at adult *L. suturalis* infested (beetle present) and non-infested (beetle absent) sites in November 2018. Comparison of main compounds

contributing to the observed differences in emissions between sites selected through SIMPER for samples collected on (a) November 2018 and (b) December 2018. Comparisons performed using the Wilcoxon rank sum test (n = 8 for each treatment). **Abbreviations:** (*E*)-2-hexanal (E2Hnal), (*Z*)-3-hexenyl 2-methylbutyrate (Z3H2M), (*E*)-4,8-dimethyl-1,3,7-nonatriene (EDMNT), Germacrene D (GerD), (*E*,*E*)-α-farnesene (aFar), (*Z*)-3-hexenyl acetate (Z3Ha), (*Z*)-3-hexenol (Z3Hnol), (*Z*)-3-hexenal (Z3Hnal), (*Z*)-3-hexenyl butyrate (Z3Hbut), (*Z*)-β-ocimene (Zbo), (*E*)-β-caryophyllene (EbC)

Table S3. List of compounds identified from the headspace of heather during the herbivory and UV experiments

		**Experiment**	
**Compound**	**Chemical class**	**Herbivory**	**UV-radiation**
(*E*)-2-hexanal	Fatty acid derivative	+	-
(*E*)-2-hexenyl acetate	Fatty acid derivative	+	-
(*Z*)-2-hexenol	Fatty acid derivative	+	-
(*Z*)-3-hexenal	Fatty acid derivative	+	+
(*Z*)-3-hexenol [i]	Fatty acid derivative	+	+
(*Z*)-3-hexenyl 2-methylbutyrate	Fatty acid derivative	+	+
(*Z*)-3-hexenyl acetate [i]	Fatty acid derivative	+	+
(*Z*)-3-hexenyl benzoate	Fatty acid derivative	+	-
(*Z*)-3-hexenyl butyrate	Fatty acid derivative	+	+
(*Z*)-3-hexenyl hexanoate	Fatty acid derivative	+	-
(*Z*)-3-hexenyl isobutyrate	Fatty acid derivative	+	-
(*Z*)-3-hexenyl isovalerate	Fatty acid derivative	+	-
(*Z*)-3-hexenyl valerate	Fatty acid derivative	+	-
Hexanal	Fatty acid derivative	+	-
Hexyl acetate	Fatty acid derivative	+	-
Jasmone	Fatty acid derivative	+	-
Hexanol	Fatty acid derivative	+	+
(*E*)-β-ocimene	Monoterpenoid	+	-
(*Z*)-β-ocimene	Monoterpenoid	+	-
Limonene	Monoterpenoid	+	-
Linalool [i]	Monoterpenoid	+	-
β-myrcene	Monoterpenoid	+	-
α-pinene [i]	Monoterpenoid	+	-
Epoxylinalol	Monoterpenoid	+	-
β-pinene [i]	Monoterpenoid	+	-
α-terpineol	Monoterpenoid	+	-

o-cymene	Monoterpenoid	+	-
α-bourbonene	Sesquiterpenoid	+	-
α-cubebene	Sesquiterpenoid	+	-
(*E*,*E*)-α-farnesene	Sesquiterpenoid	+	-
α-gurjunene	Sesquiterpenoid	+	-
α-selinene	Sesquiterpenoid	+	-
(*E*)-β-caryophyllene [i]	Sesquiterpenoid	+	-
Germacrene D	Sesquiterpenoid	+	-
Copaene	Sesquiterpenoid	+	-
γ-elemene	Sesquiterpenoid	+	-
δ-cadinene	Sesquiterpenoid	+	-
δ-guaiene	Sesquiterpenoid	+	-
Humulene [i]	Sesquiterpenoid	+	-
(*E*)-β-Farnesene	Sesquiterpenoid	+	+
(*E*)-4,8-dimethyl-1,3,7-nonatriene	Homoterpene	+	-
Benzaldehyde [i]	Aldehyde	+	+
Heptanal	Aldehyde	+	-
Nonanal	Aldehyde	+	+
Octanal	Aldehyde	+	-
Decanal	Aldehyde	+	+
(*E*)-2-nonenal	Aldehyde	+	-
Benzeneacetaldehyde	Aldehyde	-	+
Heptanol	Alcohol	+	-
Hexadecanol	Alcohol	+	-
Nonanol	Alcohol	+	-
Octanol	Alcohol	+	-
Benzyl alcohol	Alcohol	+	-
Phenylethyl alcohol	Alcohol	+	-
Geranyl nitrile	N containing compound	+	-
Indole	N containing compound	+	-

[i] Compounds verified by authentic standards

+ Compound identified

- Compound not identified
